# Neuroprotective Effects of Tauroursodeoxicholic Acid Involves Vascular and Glial Changes in Retinitis Pigmentosa Model

**DOI:** 10.3389/fnana.2022.858073

**Published:** 2022-04-12

**Authors:** Laura Fernández-Sánchez, Henar Albertos-Arranz, Isabel Ortuño-Lizarán, Pedro Lax, Nicolás Cuenca

**Affiliations:** ^1^Department of Optics, Pharmacology and Anatomy, University of Alicante, Alicante, Spain; ^2^Department of Physiology, Genetics and Microbiology, University of Alicante, Alicante, Spain; ^3^Alicante Institute for Health and Biomedical Research (ISABIAL-FISABIO Foundation), Alicante, Spain; ^4^Institute Ramón Margalef, University of Alicante, Alicante, Spain

**Keywords:** neurovascular unit, astrogliosis, neuroprotection, vascular network, neurodegenerative diseases, retina, bile acids

## Abstract

**Purpose:**

Retinitis pigmentosa is primarily characterized by a massive photoreceptor loss. But a global retinal remodeling occurs in later stages of the disease. At that phase, glial cells and retinal vasculature are also strongly affected. The main aim of the present work is to assess if the bile acid Tauroursodeoxicholic acid (TUDCA), which has a demonstrated neuroprotective effect in numerous neurodegenerative diseases, is able to prevent glial and vascular degeneration in the P23H rat retina.

**Methods:**

Homozygous P23H (line 3) animals were injected weekly with a TUDCA (500 mg/kg, i.p.) or vehicle solution, from the postnatal day (P) 21 to P120. Sprague-Dawley rats (SD) were used as control. Retinal cross-sections and wholemounts were immunostained using different glial and vascular markers and visualized with confocal microscopy. Retinal blood vessels were stained with nicotinamide adenine dinucleotide phosphate (NADPH) diaphorase histochemistry and retinal vascular networks were drawn by hand using a camera lucida.

**Results:**

At P120, the photoreceptor degeneration observed in P23H rats was accompanied by a reduction in the vascular network density and complexity at the deep capillary plexus. In addition, astrocytes showed gliotic features and the outer processes of Müller cells displayed an aberrant distribution in ring-shaped structures. When treated with TUDCA, P23H rats displayed better-preserved vessels and capillary loops in the deep capillary plexus which are associated with the partial preservation of photoreceptors. TUDCA treatment also increased the number of astrocytes and reduced the presence of Müller cell process clusters in the outer retina.

**Conclusion:**

This work suggests that, besides its neuroprotective effect on photoreceptor cells, TUDCA treatment also protects from vascular and glial degeneration, a fact that encourages the use of TUDCA as a powerful therapy for neurodegenerative diseases.

## Introduction

Retinal homeostasis is crucial for assuring the adequate functioning of the retina. In this tissue, macroglial cells (astrocytes and Müller cells) are the cells responsible for maintaining homeostasis and providing the appropriate environment for the correct functioning of the retina. Müller cells are specialized retinal glial cells that are involved in ion balance, water clearance, neurotransmitter recycling, and retinal homeostasis (Reichenbach and Bringmann, 2013, 2020). Astrocyte cells are the second glial cells in the retina and are almost entirely restricted to vascularized areas (Stone and Dreher, 1987). They are mainly involved in maintaining retinal vasculature and the blood-retina barrier (BRB) and also contribute to many of the homeostatic functions attributed to Müller cells (Coorey et al., 2012; Reichenbach and Bringmann, 2020). Both glial cell types, together with microglia, pericytes, endothelial cells, and extracellular matrix components, form a functional complex called neurovascular unit–a cooperative structure that adjusts blood supply to neuronal needs. This mechanism is responsible for vascular maintenance and BRB integrity (Klaassen et al., 2013; Reichenbach and Bringmann, 2020). Facing intrinsic or extrinsic injury, retinal tissue exhibits a vast repertoire of cellular responses to minimize or neutralize the damage (Cuenca et al., 2014). Included in that response to injury or stress, macroglial cells change to an activated phenotype (or gliosis). This state may involve both beneficial and/or harmful outcomes like the release of neurotrophic and growth factors and of molecules that increase an inflammatory environment and BRB breakdown, respectively (Bringmann et al., 2006; Coorey et al., 2012; Reichenbach and Bringmann, 2020). For example, oxidative stress, inflammation, or hyperglycemia, in both aging and disease, can disrupt the integrity of the BRB and the neurovascular unit, contributing to neurodegeneration (Klaassen et al., 2013; Cuenca et al., 2014; Tarantini et al., 2017; Reichenbach and Bringmann, 2020).

Other causes of retinal injury are inherited retinal dystrophies (IRD). Among them, retinitis pigmentosa (RP) is the most frequent form of IRD, affecting 1 in 3,500 individuals. More than 990 mutations (Ran et al., 2014) in more than 60 genes^1^ have been identified as causing the disease. Although RP is first characterized by a major loss of photoreceptors, a global retina remodeling that affects neuronal and glial cells occurs after that (Jones et al., 2003; Cuenca et al., 2014). The initial photoreceptor loss generates an imbalance between oxygen supply and consumption that drives to vascular withdrawal and triggers inflammation and gliosis, leading to a reduction in vascular network and blood perfusion in patients with RP (Jauregui et al., 2018; Corazza et al., 2020). These changes are probably affecting the integrity of the neurovascular unit since BRB breakdown has been described in the inner and outer BRB of patients with RP (Newsome, 1986; Strong et al., 2017). Similar findings have been described in RP animal models (Pennesi et al., 2008; Fernández-Sánchez et al., 2018; Kim et al., 2018) in which the vascular network is partially lost at later stages of the disease (Pennesi et al., 2008; Fernández-Sánchez et al., 2018; Kim et al., 2018).

Tauroursodeoxicholic acid (TUDCA) is a bile acid that has demonstrated neuroprotective effects in different models of retinal diseases, including RP (Boatright et al., 2006; Phillips et al., 2008; Fernández-Sánchez et al., 2011; Drack et al., 2012; Noailles et al., 2014; Lawson et al., 2016; Tao et al., 2019), Leber congenital amaurosis (Zhang et al., 2012), retinal detachment (Mantopoulos et al., 2011), diabetic retinopathy (Gaspar et al., 2013; Wang et al., 2016), or ganglion cell death (Gómez-Vicente et al., 2015; Xia et al., 2015; Kitamura et al., 2019). These protective effects of TUDCA have been usually linked to its role as an antioxidant, antiapoptotic, or anti-inflammatory agent, and to its chaperone activity (Daruich et al., 2019; Kusaczuk, 2019; Han et al., 2021; Huang et al., 2022). The aim of this work is to assess the effects of TUDCA on the vascular and glial changes occurred during the neurodegenerative process in an animal model of RP.

## Materials and Methods

### Animals

Albino homozygous P23H line-3 rats were obtained from Dr. Matthew LaVail (UCSF School of Medicine). Normal Sprague-Dawley (SD) rats, provided by Harlan Laboratories (Barcelona, Spain), were used as age-matched controls. All animals were bred in a colony at the University of Alicante and maintained under controlled conditions of humidity (60%), temperature (23°C), and photoperiod (LD 12:12). Rats were fed with dry food and water *ad libitum*. All the procedures were made following the specifications approved by the Ethics Committee of the University of Alicante (UA-2013-07-22). Animal handling was carried out in agreement with current regulations on the care and use of laboratory animals (EU Directive 2010/63/EU, NIH-guidelines, and the ARVO Statement for the Use of Animals in Ophthalmic and Vision Research).

### Tauroursodeoxicholic Acid Treatment

Tauroursodeoxicholic acid was obtained from Calbiochem (Calbiochem, Merck Millipore, Darmstadt, Germany) and dissolved in phosphate-buffered saline (PBS, pH 7.4) by using an ultrasonic bath to avoid bubble formation. Treatment was performed as previously described by our group (Fernández-Sánchez et al., 2011; Noailles et al., 2014). Briefly, P23H line-3 animals were divided into two groups: TUDCA-treated and vehicle-treated animals. The TUDCA-treated group (*n* = 12) was administered with TUDCA (500 mg/Kg, i.p.) once a week from postnatal day (P)21 to P120. Vehicle-treated P23H rats (*n* = 15) were administered with a vehicle at the same time points. SD animals (*n* = 11) were administered with the vehicle. At the end of the treatment, all animals were sacrificed, and tissue was processed.

### Retinal Histology

Animals were sacrificed between 10:00 a.m. and 12:00 p.m. by administering a lethal dose of sodium pentobarbital (200 mg/kg). Prior to eye enucleation, a suture was sewn in the dorsal limbus to label axis orientation. After enucleation, an incision was made at the sclerocorneal limbus to allow better penetration of the fixative solution. The eyecups were immersed in paraformaldehyde 4% (w/v) and PBS for 1 h. After that, eyes were washed three times in PBS and cryoprotected with increasing concentration of sucrose solutions: 15% (1 h), 20% (1 h), and 30% (overnight). The posterior pole of the eye was isolated by removing the cornea, lens, and vitreous body, and processed for nasal-temporal retinal cross-sections or wholemounts. In most cases, whenever possible, one retina from each animal was prepared as a whole mount and the other was used for sections. For whole mount preparations, retinas were carefully isolated from the rest of the eyecup by using a fine brush. Four partial incisions were made along the retinal axis to divide the retinas into superior, inferior, nasal, and temporal quadrants, and to better flatten them. Retinal wholemounts were finally mounted with the ganglion-cell-side up. For sections, the eyecups were embedded in optical coherence tomography (OCT) and frozen in liquid nitrogen. Sixteen-micrometer-thick cross-sections were obtained using a Leica CM 1900 cryostat (Leica Microsystems, Wetzlar, Germany). Sections were mounted on slides (Superfrost Plus; Menzel GmbH and Co., KG, Braunschweig, Germany) and air-dried. Prior to further processing, the slides were washed in PBS and treated for 1 h using blocking solution (10% normal donkey serum in PBS plus 0.5% TritonX-100).

### Retinal Immunohistochemistry

Retinas from all groups were processed in parallel to objectively compare them. The cell-specificity of all antibodies and lectins used in this word has been well characterized by our group and others and has been used extensively in other studies. Staining of retinal wholemounts and sections was performed following the standard protocols previously described (Fernández-Sánchez et al., 2015a). Briefly, retinal tissue was washed three times in PBS and then incubated in 0.02% sodium borohydride (163314; Panreac, Barcelona, Spain) in PBS (5 min, RT) to increase permeability to the antibodies. Sections and whole mount retinas were incubated with a combination of primary antibodies at an optimal concentration (Table 1) at 4°C overnight or for 3 days, respectively. Then, sections or whole mount retinas were washed three times in PBS and incubated for 1 h or overnight, respectively, with the corresponding Alexa fluor-conjugated antibody or secondary antibody and *Griffonia simplicifolia* IB_4_ (1:100, I21411; Isolectin GS-IB_4_, Invitrogen, Carlsbad, CA, United States) at the optimal concentration. The secondary antibodies used in this work were Alexa Fluor 488-conjugated anti-rabbit IgG, Alexa Fluor 555-conjugated anti-mouse IgG, and Alexa Fluor 555-conjugated anti-rabbit IgG made in donkey, and all of them were obtained from Molecular Probes (Eugene, OR, United States). After washing, the preparations were mounted in Citifluor (Citifluor Ltd, London, United Kingdom) and coverslipped for further observation under a laser-scanning confocal microscopy Leica TCS SP2 (Leica Microsystems, Wetzlar, Germany).

**TABLE 1:** Antibodies used for immunofluorescence.

Molecular marker	Antibody	Supplier (Catalog No.)	Dilution
Recoverin	Mouse monoclonal	J.F. McGinnis, University of Oklahoma, United States	1:2000
Transducin, Gac subunit	Rabbit, polyclonal	CytoSignal	1:200
Glial fibrillary acidic protein (GFAP)	Mouse, G-A-5	Sigma (G3893)	1:500
Glial fibrillary acidic protein (GFAP)	Rabbit, polyclonal	Dako (N1506)	1:50
Vimentin	Mouse, V9	Dako (M0725)	1:100

*Supplier locations: CytoSignal (San Diego, CA, United States); Sigma (St. Louis, MO, United States); Dako (Glostrup, Denmark).*

### Vascular Network Quantification

The retinal vascular network was visualized using histochemistry of reduced nicotinamide adenine dinucleotide phosphate diaphorase (NADPH-d) as previously described by our group (Fernández-Sánchez et al., 2018). In brief, whole mount retinas were incubated in PBS containing 1 mg/ml NADPH (Sigma, St. Quentin Fallavier, France), 0.1 mg/ml nitroblue tetrazolium (NBT) (Sigma), and 1% (v/v) Triton X-100 for 2 h at 37°C. After washing three times in PBS, retinas were flat-mounted in Citifluor mounting medium (Citifluor Ltd.) with ganglion-cell-side up. Deep vascular plexus was drawn using a camera lucida coupled to a Leica DMR microscope (Leica Microsystems, Wetzlar, Germany). Drawings were digitalized and morphometric analysis was performed using ImageJ software (National Institutes of Health, Bethesda, MD, United States) and AngioTool software (0.6a version, National Cancer Institute, Bethesda, MD, United States) (Zudaire et al., 2011). Prior to analysis, Adobe Photoshop software (Adobe Systems, Inc., San Jose, CA, United States) was used to equally adjust brightness and contrast on the images. ImageJ software was used to measure the relative capillary density of the deep capillary plexus (DCP) with the “measure area” function, and the total number of capillary loops was studied using the “analyze particles” command. Angio-tool software (0.6a version, National Cancer Institute, Bethesda, MD, United States) was used to study in detail the vascular network complexity. Specifically, vessel junction density (which indicates the vascular branching per unit area), total vessel length, and mean lacunarity were analyzed. Lacunarity measurements describe the non-uniformity of the vascular pattern. It offers information about the size of lacunas or gaps present in the images (Gould et al., 2011; Zudaire et al., 2011). Thus, greater lacunarity means greater gaps and vascular degeneration. All data were normalized to the retinal area.

### Astrocyte Quantification

Astrocyte quantification was done as previously described by our group (Fernández-Sánchez et al., 2015a). Briefly, astrocytes were quantified in whole mount retinas immunolabelled against the glial fibrillary acidic protein (GFAP) and nuclear marker TO-PRO-3. Lectin GS-IB_4_ was used to visualize retinal blood vessels. Quantifications were done in 12 representative areas measuring 0.227 mm^2^ and homogeneously distributed in the superior, inferior, nasal, and temporal quadrants from retinal center to the periphery. Astrocyte cell bodies were manually quantified in each region in two confocal images, and averaged values were used for each area. Only GFAP-positive cells with a well-defined nucleus were included in the counting. At least three rats were used per data point.

### Statistical Analysis

Prism software (GraphPad Software; San Diego, CA, United States) was used to perform statistical analysis. One- or two-way ANOVA tests were applied to evaluate the effects of TUDCA on morphological parameters of the vascular network and astrocyte numbers. Normal distributions and homogeneity of variance were found for all the analyzed categories. *p* < 0.05 was considered statistically significant. When the level of significance was 0.05 or less, *post-hoc* pairwise comparisons using Tukey’s test were carried out. Data were plotted representing the mean ± standard error of the mean (Osipova et al., 2018).

## Results

### Tauroursodeoxicholic Acid Preserves Both Retinal Photoreceptors and Retinal Vasculature

**FIGURE 1:**
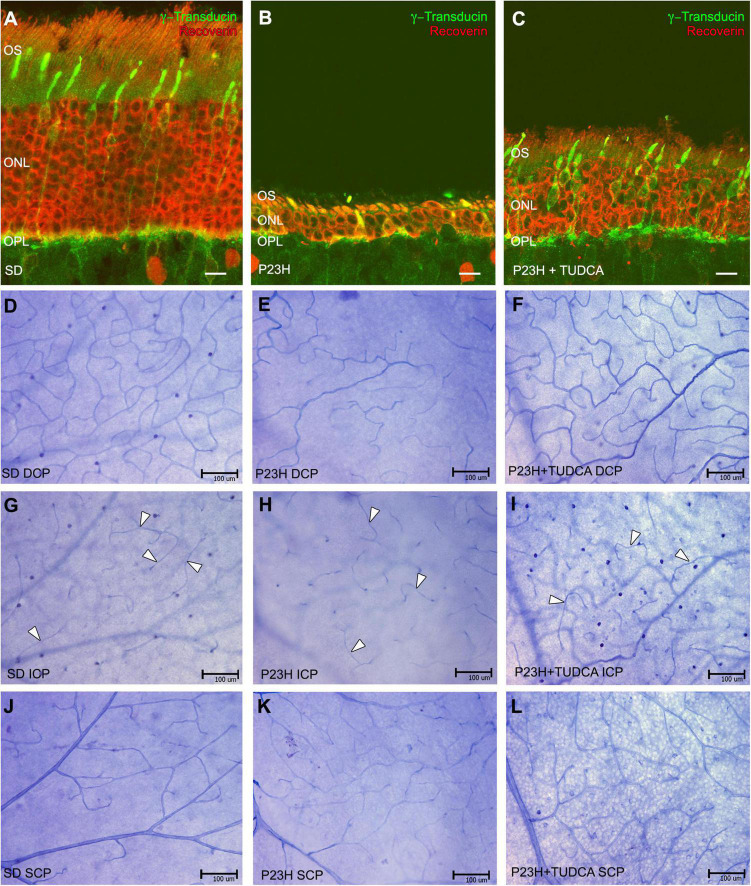
Tauroursodeoxicholic acid (TUDCA) effects on vascular changes associated with photoreceptor death. **(A–C)** Retinal cross-sections stained with antibodies against recoverin (red) and γ-transducin (green) in a Sprague-Dawley rat (SD) **(A)**, P23H **(B)**, and TUDCA-treated P23H **(C)** rat at postnatal day (P) 120. **(D–L)** Whole mount retinas from an SD **(D,G,J)**, P23H **(E,H,K)**, and TUDCA-treated P23H **(F,I,L)** rat at P120 stained with Nicotinamide Adenine Dinucleotide Phosphate (NADPH) diaphorase. All images were taken from the medial areas of the retina avoiding the major blood vessels. P23H retinas showed an evident loss of photoreceptors **(B)**, accompanied by degenerated deep capillary plexus (DCP) **(E)** compared to SD animals **(A,D)**; TUDCA was able to reduce these changes **(C,F)**. No differences between groups were observed at intermediate capillary plexus (ICP) **(G–I)** and superficial capillary plexus (SCP) **(J–L)**. Note that the ICP showed less dense but well-defined vessels (arrowheads) in all animals studied. OS, outer segments; ONL, outer nuclear layer; OPL, outer plexiform layer; DCP, deep capillary plexus; ICP, intermediated capillary plexus; SCP, superficial capillary plexus. *Scale bars*: 10 μm **(A–C)**, 100 μm **(D–L)**.

Retinal vasculature in rodents is distributed in three laminar plexuses: the superficial capillary plexus (SCP) at the level of the ganglion cell layer (GCL), the DCP at the level of the outer plexiform layer (OPL), and the intermediate capillary plexus (ICP) which connects the other two, running at the edge between the inner nuclear (INL) and inner plexiform (IPL) layers. Figure 1 shows the state of photoreceptor cell death and vascular network degeneration at each of the retinal capillary plexuses in P120 rats from the different experimental groups. Retinal sections from SD rats showed 10–12 rows of photoreceptors at the outer nuclear layer (ONL) and normal photoreceptor morphology (Figure 1A). In those animals, NADPH-d staining revealed the typical density and distribution of capillary loops in the DCP (Figure 1D), well-defined arteries and veins at the SCP (Figure 1J), and a less dense but well-defined and continuous capillary network at the ICP (Figure 1G, arrowheads). In P23H rat retinas, an advanced degeneration of the ONL occurs at that age, where only a couple of photoreceptor cell rows remain and there are evident morphological alterations in both rods and cones (Figure 1B). Besides the photoreceptor degeneration, we also observed a reduction in the capillary content in the DCP with evident loss of capillary loops (Figure 1E). Nevertheless, the intermediate and superficial vascular plexuses showed their typical structure in these rats (Figures 1H,K, respectively). When TUDCA treatment was administered, it was able to diminish the loss of photoreceptor cells and preserve their morphology. Hence, cones and rods present longer outer segments and normal terminal axons (Figure 1C). In addition, it protected vascular degeneration, and treated animals displayed a better-preserved capillary network. The DCP of TUDCA-treated P23H rats (Figure 1F) showed more vessels and more capillary loops compared to vehicle-treated P23H rats. No differences were found in the ICP and SCP (Figures 1I,L, respectively).

### Tauroursodeoxicholic Acid Preserves Retinal Deep Capillary Plexus Density and Complexity

**FIGURE 2:**
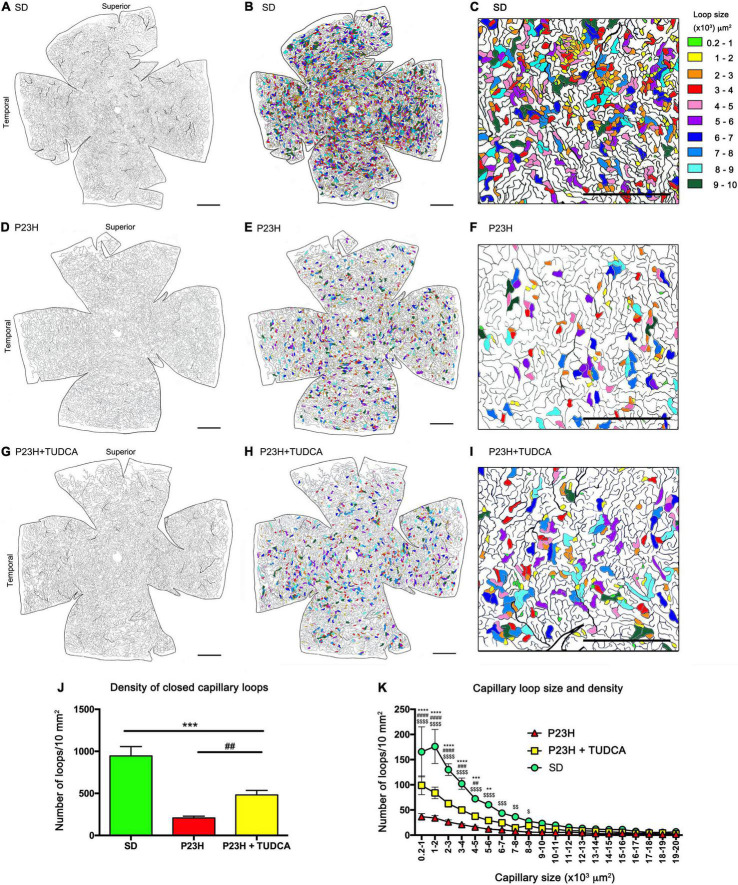
Tauroursodeoxicholic acid (TUDCA) effects on degenerative changes in the deep capillary plexus. **(A–I)** Representative drawings of the DCP vessels from an SD **(A–C)**, P23H **(D–F)**, and TUDCA-treated P23H **(G–I)** rat at P120. The retinal area circumscribed by each enclosed capillary loop had been labeled in different colors according to their size **(B,E,H)**. Magnifications **(C,F,I)** correspond to the medial region of the temporal retina. The images showed a marked reduction in the number of closed capillary loops in P23H compared to SD retinas. These differences are less evident in retinas from TUDCA treated P23H animals. **(J)** Mean density of closed capillary loops in the DCP of SD, P23H, and TUDCA-treated P23H rats at P120. Note the high decrease in the number of closed loops in P23H retinas and the protective effect of TUDCA. **(K)** Relationship between closed capillary loop size and density in the same retinas than in **(J)**. Note that the relative loss of closed capillary loops in P23H rats was inversely proportional to the loop size. One-way **(J)** and two-way **(K)** ANOVA, Tukey’s multiple comparison test; TUDCA-treated P23H vs. SD: **p < 0.01, ***p < 0.001, ****p < 0.0001; TUDCA-treated vs. vehicle-treated P23H: ^##^*p* < 0.01, ^###^*p* < 0.001, ^####^*p* < 0.0001; vehicle-treated P23H vs. SD: ^$^*p* < 0.05, ^$$^*p* < 0.01, ^$$$^*p* < 0.001, ^$$$$^*p* < 0.0001. SD, *n* = 3; P23H, *n* = 7; P23H + TUDCA, *n* = 6. *Scale bars*: 1 mm.

To assess in more detail the effects of TUDCA on capillary plexus, blood vessels at the DCP throughout the entire retina were drawn and digitalized, and DCP vessel density and complexity were analyzed. Figure 2 shows the drawing of the entire retinal DCP of P120 rats from the three experimental groups. The area occupied by closed capillary loops was colored according to the size of the loops whose areas ranged from 200 to 10,000 μm^2^. The density of the DCP in SD retinas (Figure 2A) and closed capillary loops of different sizes (Figure 2B) showed a typical and homogeneous distribution through the entire retina where small size loops were numerous (Figures 2C,K). TUDCA-treated P23H retinas showed less deteriorated DCP (Figure 2G) compared to vehicle-treated P23H retinas, in which a remarkable reduction of capillary density, affecting more extensively the superior and temporal quadrants, was detected (Figure 2D). This plexus degeneration was also revealed by the decrease in closed capillary loops density by 78% (Figures 2E,J), which predominantly affects the smallest capillary loops (Figures 2F,K). In these animals, TUDCA treatment resulted in a less deteriorated DCP in which closed capillary loops, even the smallest ones, were more abundant (Figures 2H–K) than in the vehicle-treated P23H retinas (Figures 2E,F).

**FIGURE 3:**
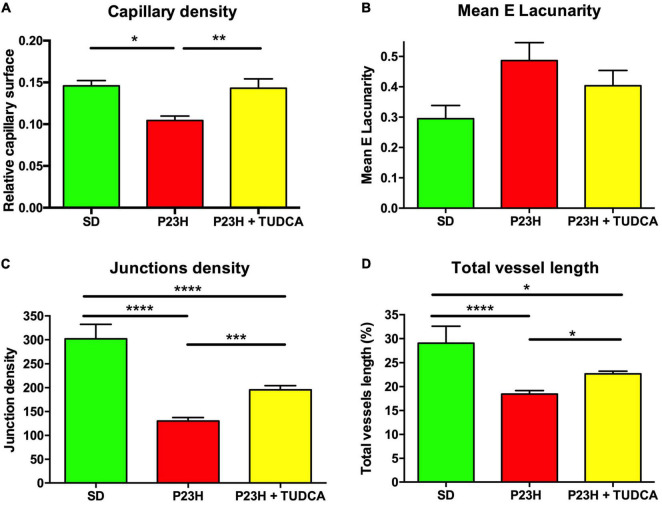
Tauroursodeoxicholic acid (TUDCA) effects on deep capillary plexus density and complexity of P23H rats. **(A)** Mean relative capillary density (capillary area/retinal area), **(B)** mean E lacunarity values, **(C)** mean junction density, and **(D)** mean percentage of blood vessel length, for the DCP in SD, P23H, and TUDCA-treated P23H rats at P120. One-way ANOVA, Tukey’s multiple comparison test; *p < 0.05, **p < 0.01, ***p < 0.001, ****p < 0.0001. SD: *n* = 3, P23H: *n* = 7, P23H + TUDCA: *n* = 6.

Additional quantitative analysis of the DCP showed a decrease of 28% in the relative capillary surface area (vascular area/retinal area) of P23H compared to SD retinas (Figure 3A). TUDCA treatment was able to partially avoid this loss of DCP vessels. The capillary surface of DCP in the TUDCA-treated group was significantly higher (37%) than in vehicle-administered P23H rats (Figure 3A). No significant differences were observed between TUDCA-treated P23H retinas and SD retinas. In addition to the capillary density, the mean lacunarity was also calculated as a measure of the vascular network complexity. Although no significant differences were found between groups, lacunarity values were relatively higher in P23H retinas than in TUDCA-treated retinas and SD retinas (Figure 3B). Greater lacunarity indicates the presence of more gaps or lacunas. Thus, this tendency suggests a reduction in vascular complexity of dystrophic P23H retinas and a protective effect of TUDCA. The reduction in vascular complexity of dystrophic animals was ratified by a significant decrease in vessel junction density in P23H retinas as compared to SD retinas, indicating a reduction in vascular branching complexity (Figure 3C). Vessel junction density in TUDCA-treated P23H retinas was significantly higher than that measured in vehicle-treated dystrophic animals (Figure 3C), confirming the protective effects of TUDCA. Similar effects were observed in the percentage of total vessel length as observed in how vehicle-treated P23H rats showed lower values than TUDCA-treated animals. In addition, both groups presented a reduced vessel length compared to SD rats (Figure 3D). These results indicate that DCP of P23H rats has a more irregular vascular pattern with larger gaps and, overall, greater vascular degeneration that can be partially prevented by TUDCA administration.

### Tauroursodeoxicholic Acid Affects Astrocyte Morphology and Density

**FIGURE 4:**
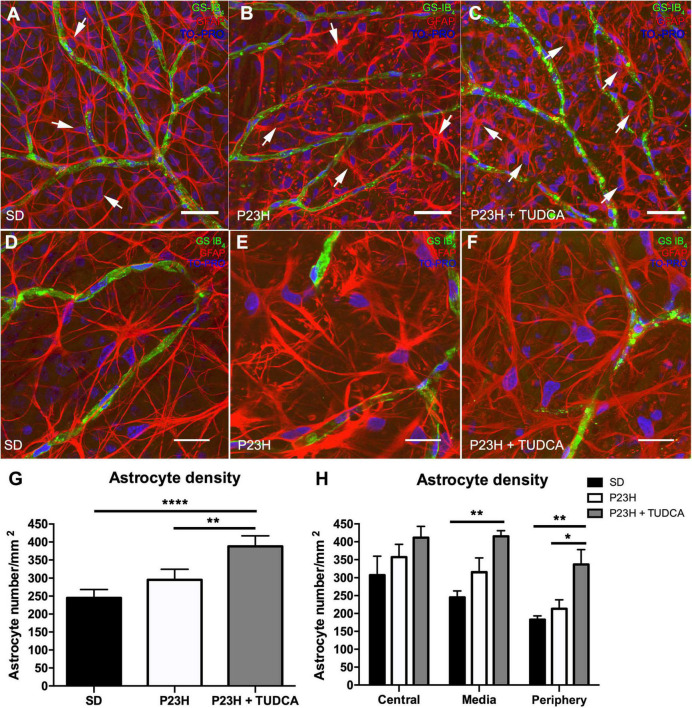
Tauroursodeoxicholic acid (TUDCA) effects on retinal astrocyte density, distribution, and morphology in P23H rats. Whole mount retinas from an SD **(A)**, P23H **(B)**, and TUDCA-treated P23H **(C)** rat at P120, showing the distribution of retinal astrocytes (Glial Fibrillary Acidic Protein, GFAP; red, arrows) associated with SCP vessels (isolectin GS-IB_4_; green) at the nerve fiber layer. Nuclei were stained with TO-PRO 3 iodide (blue). Images were taken from the medial retina. Note the presence of GFAP-positive Müller cell end-feet in vehicle- and TUDCA-treated P23H retinas **(B,C)**. **(D–F)** High magnification view of astrocytes (GFAP, red) and SCP vessels (isolectin GS-IB_4_; green). Note that astrocytes in P23H retinas **(E)** show thickened processes and gliotic features (increased staining for GFAP and hypertrophy) compared to SD retinas **(D)**, and that TUDCA treatment **(F)** reduce these changes. **(G)** Mean retinal astrocyte density in the three experimental groups. **(H)** Mean density values in the central, medial, and peripheral retina. Scale bare: 40 μm **(A–C)** and 20 μm **(D–F)**. One-way **(G)** and two-way **(H)** ANOVA, Tukey’s multiple comparison test; *p < 0.05, **p < 0.01, ****p < 0.0001. SD: *n* = 3, P23H: *n* = 3, P23H + TUDCA: *n* = 3.

To assess the effect of TUDCA treatment in the glial response of P23H rat retinas, the morphology and density of astrocytes were studied. As shown in Figure 4, astrocyte cell bodies were located at the ganglion cell layer and homogeneously distributed throughout the whole retina in all groups studied (Figures 4A–F; arrows). In all the animals, astrocytes displayed their typical flattened morphology with radial and longitudinal processes extending from the cell body to other astrocytes or blood vessels (Figure 4D). In P23H retinas, astrocytes presented gliotic features with hypertrophy of astrocytic processes (Figure 4E) compared to SD retinas (Figure 4D). In contrast, TUDCA-treated retinas (Figure 4F) showed milder gliosis, and the hypertrophy of the astrocytic processes was less evident than in vehicle-treated P23H retinas, suggesting that TUDCA partially prevents strong astrocyte gliosis.

Astrocyte quantification revealed a small, non-significant, increase in astrocyte density of P23H retinas that was exacerbated in TUDCA-treated retinas, in which astrocyte density was significantly higher than in vehicle-treated P23H and SD retinas (Figure 4G). Quantification of astrocytes in the central, medial, and peripheral retina showed a global TUDCA-induced increase in astrocyte density (Figure 4H), with greater differences in the peripheral retina. GFAP immunoreactive spots corresponding to end-feet of Müller cells were easily recognizable in TUDCA-treated and vehicle-treated P23H retinas (Figures 4B,C), suggesting Müller cell gliosis in those animals.

### Tauroursodeoxicholic Acid Influences Gliosis and Morphology Changes in Müller Cells

**FIGURE 5:**
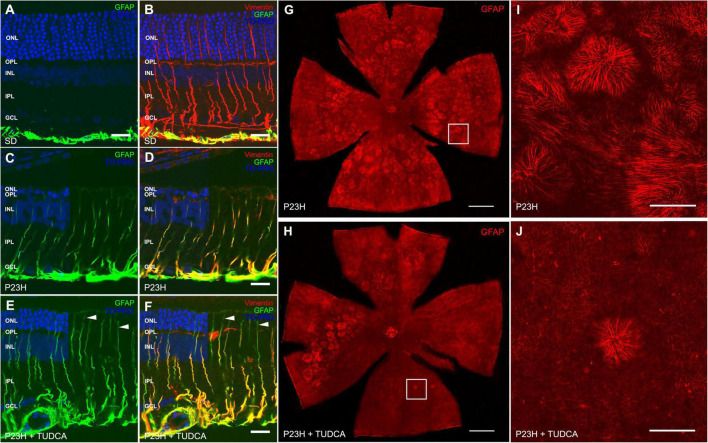
Tauroursodeoxicholic acid (TUDCA) effects on Müller cell gliotic changes in P23H rats. **(A–F)** Retinal cross-sections from an SD **(A,B)**, P23H **(C,D)**, and TUDCA-treated P23H **(E,F)** rat at P120, immunostained against GFAP (green) and vimentin (red). TO-PRO 3 iodide was used to visualize the nuclei (blue). All images were collected from the medial retina. Note the increased immunoreactivity against GFAP in Müller cells from dystrophic retinas, indicating reactive gliosis. No remarkable effects of TUDCA are observed. **(G,H)** Whole mount retinas from a vehicle-treated **(G)** and TUDCA-treated **(H)** P23H rat at P120 immunostained against GFAP, showing the distribution of the apical processes of Müller cells in the ONL. Only representative dystrophic rats are included in the figure since only gliotic Müller cells express GFAP. **(I,J)** High magnification of apical processes of Müller cells at the outer retina. Note the presence of Müller cell processes arranged in clusters forming firework-like structures, more numerous in vehicle-treated than in TUDCA-treated P23H retinas. A minimum of *n* = 5 animals per group were analyzed. ONL: outer nuclear layer; OPL: outer plexiform layer; INL: inner nuclear layer; IPL: inner plexiform layer; GCL: ganglion cell layer. Scale bar: 20 μm **(A–F)**, 1 mm **(G,H)**, 200 μm **(I,J)**.

To better analyze the effects of TUDCA on retinal gliosis, we performed immunostaining against two intermediate filaments, GFAP and vimentin, in retinal cross-sections from the three experimental groups (Figures 5A–F). In SD retinas, GFAP immunostaining was only present in astrocytes (Figures 5A,B, green), while vimentin immunolabels both astrocytes and Müller cells (Figure 5B, red). In P23H rat retinas, vimentin immunostaining was similar to that observed in SD retinas, independent of the treatment (Figure 5D). However, Müller cells were highly positive to GFAP immunolabeling in both TUDCA-treated and vehicle-treated P23H rats, indicating extensively gliosis of Müller cells in dystrophic animals (Figures 5C,F, green) even if no differences in the number of Müller cells were detected between all groups (data not shown). GFAP staining was observed at the ONL level in the cross-sections of the TUDCA group since photoreceptors are preserved and Müller cell processes reach the external limiting membrane (Figures 5E,F, arrowheads). The loss of photoreceptor rows in vehicle-treated P23H rats resulted in less appreciable Müller cell apical processes at the ONL.

To assess TUDCA effects on the outer Müller cell processes of dystrophic rats, whole mount retinas from vehicle- and TUDCA-treated animals were immunolabeled with antibodies against GFAP, and confocal images were taken at the ONL (where the outer end of Müller processes is located) (Figures 5G–J). At this level, clusters of hypertrophied processes of Müller glia forming ring-shaped firework-like structures were numerous and widely distributed through the central and medial parts of the superior, inferior, nasal, and temporal quadrants of the retina of P120 vehicle-treated P23H rats (Figures 5G,I). In this animal model, the photoreceptor loss causes the outer end of Müller cells to laterally fall with the remnants of the ONL, developing these structures (García-Ayuso et al., 2013; Fernández-Sánchez et al., 2015a). Clusters of Müller cell processes were numerous and widely distributed through the central and medial parts of the superior, inferior, nasal, and temporal quadrants of the retina of P120 vehicle-treated P23H rats (Figures 5G,I). TUDCA decreased the presence of firework-like structures which were only visible in the medial part of the temporal and superior quadrants of the retina of treated animals (Figures 5H,J). The lack of these structures is due to the preservation of photoreceptors at the ONL.

## Discussion

Retinal degeneration in RP triggers a set of cell signals that drives retinal remodeling, including glial and vascular changes (Jones et al., 2003; Lewis and Fisher, 2003; Cuenca et al., 2014). Previously published data by our group showed that treatment with the bile acid TUDCA is able to ameliorate retinal degeneration in P23H rats (Fernández-Sánchez et al., 2011; Fernández-Sánchez et al., 2015b; Fernández-Sánchez et al., 2017) and to reduce retinal inflammation by decreasing microglial activation (Noailles et al., 2014). The present study shows that TUDCA treatment can also be protective by partially preventing the vascular damage and glial activation previously described in this RP rat model (Fernández-Sánchez et al., 2015a; Fernández-Sánchez et al., 2018).

In the recent years, a relationship between pathological traits of neurodegenerative diseases and altered bile acid metabolism has been described (Nho et al., 2019; Bhargava et al., 2020), and increasing data supporting the neuroprotective effects of bile acids have been reported (Ackerman and Gerhard, 2016; Daruich et al., 2019). In line with these data, our results show that TUDCA treatment reduces vascular degeneration associated with the progression of the disease. One of the main reasons for DCP loss in patients with RP (Jauregui et al., 2018; Corazza et al., 2020) and animal models (Pennesi et al., 2008; Cuenca et al., 2014; Fernández-Sánchez et al., 2018) is thought to be the imbalance between oxygen delivery and consumption caused by photoreceptors degeneration, which generates an increase in oxygen tension in the outer retina (Yu et al., 2000; Yu and Cringle, 2005). The protective effects of TUDCA described in this work could be strictly related to its capability of protecting photoreceptor cells. By maintaining photoreceptor cell survival, DCP integrity would also be preserved (Fernández-Sánchez et al., 2012). However, other direct mechanisms could be implicated, as multiple studies have described different effects of TUDCA on vascular endothelial cells. *In vitro* studies have shown that TUDCA is able to decrease the oxidative stress triggered under hyperglycemic conditions in a human retinal microvascular endothelial cell line (Wang et al., 2016), and to reduce endothelial dysfunction by lowering endoplasmic reticulum stress and its derivative oxidative stress in primary cultures of endothelial cells (Galán et al., 2014; Lenin et al., 2018). Also, TUDCA can repair blood vessels by supporting endothelial progenitor cell mobilization and integration, and by diminishing their senescence and oxidative stress (Cho et al., 2015). Besides, TUDCA can decrease the expression of endothelial inflammatory molecules as Intercellular Adhesive Molecule 1 (ICAM-1), nitric oxide synthase (NOS), and vascular endothelial growth factor (VEGF) in the retina of diabetic rats (Wang et al., 2016).

In addition to the vascular changes, activation of retinal astrocytes and Müller glia in response to retinal injury has also been described in RP (Fernández-Sánchez et al., 2015a), other retinal diseases (Bringmann et al., 2006; de Hoz et al., 2016), and neurodegenerative diseases affecting other parts of the central nervous system (CNS) (Li et al., 2019). Our results show that astrocyte number in P23H retinas increases with TUDCA treatment, although their morphology and GFAP staining intensity seems less gliotic than in vehicle-treated P23H retinas. The functional meaning of these findings is controversial because of the large number of evidence supporting both harmful and beneficial effects of astrogliosis. In fact, two differentiated phenotypes of reactive astrocytes have been described in neurodegeneration. Particularly, type A1 astrocytes have a neurotoxic effect due to an increased release of pro-inflammatory molecules, while type A2 astrocytes display a neuroprotective activity due to an increased release of neurotrophic factors (Li et al., 2019). Therefore, it seems that the ability of astrocytes to promote a neurotoxic or neuroprotective environment lies more in the type of astrocyte that participates in astrogliosis rather than in the number of astrocytes involved. In this sense, Bhargava and collaborators found that *in vitro* TUDCA treatment on mouse astrocyte cells was able to prevent neurotoxic polarization of astrocytes to the A1 phenotype and to reduce microglial activation, mainly through the activation of a G-protein coupled bile acid receptor (GPBAR1) (Bhargava et al., 2020). A possible hypothesis to explain our results could be related to that beneficial effect of TUDCA on astrocyte phenotype. In that sense, even if we see an increased number of astrocytes in the TUDCA-treated retinas, they could reflect the proliferation of anti-inflammatory A2 astrocytes and thus have a neuroprotective effect in retinal and vascular degeneration. Although some of the above-described research suggests that this is a possible hypothesis, it would need further validation in the specific context of our research.

Müller cells are the main macroglial cell type in the retina and, along with astrocytes, are involved in gliosis and retinal remodeling (Bringmann et al., 2006; Cuenca et al., 2014) associated with photoreceptor cell loss in RP. When rod photoreceptors die, outer Müller cell processes appear distributed in clusters, forming ring-shaped firework-like structures containing the last surviving cone photoreceptors. The number of ring-shaped structures through the outer retina increases during the course of degeneration (Lee et al., 2011; García-Ayuso et al., 2013; Fernández-Sánchez et al., 2015a). Our results show that TUDCA treatment is able to decrease the number of these structures, probably by anti-inflammatory mechanisms and by reducing the photoreceptor cell loss.

Astrocytes, Müller glia, and vascular endothelial cells are the components of the retinal neurovascular unit. This functional structure is mainly responsible for the formation and maintenance of the BRB, which ensures the selective traffic of molecules from the blood to preserve retinal homeostasis and to provide a healthy environment for neuronal cell function (Klaassen et al., 2013; Phipps et al., 2019). Communication between cells of the neurovascular unit is achieved through two main mechanisms, namely, the release of gliotransmitters and the astrocyte coupling by gap junctions (Metea and Newman, 2007; Klaassen et al., 2013; Osipova et al., 2018; Reichenbach and Bringmann, 2020), which are mainly mediated by Cx43 (Nagy and Rash, 2000; Zahs et al., 2003; Kerr et al., 2010). Pathological mechanisms, such as oxidative stress, inflammation, ischemia, or diabetes, can induce the breakdown of gap junctional communication (Klaassen et al., 2013; Cuenca et al., 2014). In this context, an *in vitro* study revealed that TUDCA is able to restore astrocyte communication through gap junctions in astrocyte cultures under chemical endoplasmic reticulum (ER) stress and hyperglycemia (Gandhi et al., 2010). Here, we showed an increased number of astrocytes in TUDCA treated retinas. Based on this, we could hypothesize that TUDCA treatment can contribute to improved functioning of the neurovascular unit in P23H retinas.

Considering that vascular and glial changes in RP are triggered by photoreceptor cell death, one hypothesis is that the protective features of TUDCA on retinal neurovascular units are exclusively due to its neuroprotective effects on photoreceptors. However, the increasing evidence of a direct effect of TUDCA in astrocytes and endothelial cells suggests that TUDCA treatment in RP could also exert direct beneficial effects on glial and vascular cells, protecting the neurovascular unit and, in turn, maintaining retinal homeostasis. Overall, our results show that the ability of TUDCA to protect retinal tissue from degeneration is broad, affecting multiple retinal cell types and preventing degeneration even at late stages of the disease.

## Data Availability Statement

The raw data supporting the conclusions of this article will be made available by the authors, without undue reservation.

## Ethics Statement

The animal study was reviewed and approved by the Ethics Committee of the University of Alicante (UA-2013-07-22).

## Author Contributions

NC, LF-S, and PL designed the experiments. LF-S performed the experiments. LF-S, HA-A, IO-L, PL, and NC analyzed the results and wrote the manuscript. All the authors approved the manuscript.

## Conflict of Interest

The authors declare that the research was conducted in the absence of any commercial or financial relationships that could be construed as a potential conflict of interest.

## Publisher’s Note

All claims expressed in this article are solely those of the authors and do not necessarily represent those of their affiliated organizations, or those of the publisher, the editors and the reviewers. Any product that may be evaluated in this article, or claim that may be made by its manufacturer, is not guaranteed or endorsed by the publisher.

## Footnotes

1
https://sph.uth.edu/retnet/home.htm
